# From a Parkinson's disease expert: Rasagiline and the Future of Therapy

**DOI:** 10.1186/1750-1326-2-13

**Published:** 2007-07-06

**Authors:** Shaheen E Lakhan

**Affiliations:** 1 Global Neuroscience Initiative Foundation, Los Angeles, CA, USA

## Abstract

John Finberg is a professor of pharmacology at the Faculty of Medicine, Technion – Israel Institute of Technology, home of Israel's two Nobel laureates. He and his colleague Prof. Moussa Youdim were instrumental in the early clinical development of the anti-Parkinson drug rasagiline, which gained UK- and EU-marketing authorization in 2005 and US FDA approval in 2006. In our interview, Finberg reflects on his clinical research to develop rasagiline as a commercial drug and its proposed pharmacological mechanisms of action. Moreover, he elucidates the current state of anti-Parkinson drug discovery and offers direction for future research.

## What focus have you taken in the field of pharmacology, and what interesting results have you seen?

Since joining the Faculty of Medicine at the Technion, Haifa, most of my research has been connected with catecholamines, mainly studies on the mechanism of the action of antidepressants, and on drugs for the treatment of Parkinson's disease. My major interest has been on the pharmacology of MAO inhibitors. These are fascinating compounds, since they can be used to cause irreversible and highly selective inactivation of one of the isoforms, i.e., MAO-A or MAO-B. Because they cause "laser-like" selective enzyme inactivation, one can use them to study not only the pharmacological effects of the drugs, but also the physiological role of these important enzymes. Inhibitors of the A form of the enzyme are effective antidepressants, and I was interested to understand the way in which inhibition of this enzyme affects neuronal noradrenaline release. Remember that, like other neurotransmitters, noradrenaline is released physiologically by exocytosis, and cleared from the extracellular space mainly by reuptake, so the effects of MAO inhibition are not easily predictable. Using in vivo micro dialysis, I was able to show that long-term administration of MAO-A inhibitors does increase CNS extracellular noradrenaline levels [1], by reduction in *net *neuronal uptake, and a similar effect occurs in the periphery.

I was also interested in the pharmacology of MAO-B inhibitors. My colleague Professor Moussa Youdim observed selective MAO-B inhibitory property in a compound now known as rasagiline. The only other selective MAO-B inhibitor available for clinical use before the advent of rasagiline was selegiline, a compound based on the amphetamine structure. The important questions at the start of this project were (a) is the nonpotentiation of tyramine by selegiline due to its amphetamine-like properties, and (b) will rasagiline increase striatal dopamine levels, in spite of its lack of amphetamine-like effect? With graduate students Meir Tenne and Itschak Lamensdorf, we showed that nonpotentiation of tyramine is a general property of MAO-B inhibitors (because MAO-A is the enzyme isoform expressed in sympathetic neurons). We also showed, using in vivo micro dialysis, that both rasagiline and selegiline increased extracellular dopamine levels in rats, provided they are given chronically [2,3]. These studies paved the way for the clinical development of rasagiline for treatment of Parkinson's disease. Subsequently, with colleague Dr. John Commissiong (NIH) and graduate student Daphna Bonneh-Barkay, we observed the neuroprotective properties of rasagiline [4,5].

## What is the proposed mechanism for the anti-Parkinson activity of rasagiline?

Rasagiline, like selegiline, has anti-parkinsonian activity when used in monotherapy in the early stages of the disease. It has always been difficult to understand how selective inhibition of MAO-B causes this apparent dopaminergic response, since MAO-A is thought to be the major form responsible for dopamine breakdown in the intact striatum [see reference [6] for a detailed discussion]. One explanation for the effectiveness of MAO-B inhibitors to increase dopamine release could be the accumulation of endogenous β-phenylethylamine. This interesting dopamine-releasing "trace amine" is normally very rapidly metabolized by MAO-B, but its concentration increases markedly with inhibition of the enzyme. In addition, primate species express a much higher proportion of MAO-B to MAO-A activity in the brain than do rodents. Currently, I am studying how MAO-B inhibitors affect the metabolism of dopamine, both that released endogenously, and also from exogenous L-DOPA, and how this process changes with progressive loss of dopaminergic neurons, and increasing metabolism in glia.

## Where does rasagiline fit into the current scope of Parkinson's disease therapy?

Rasagiline is effective in monotherapy in the early stage of the disease, and can be used instead of dopaminergic agonists. In the more advanced stages of the disease, it is given together with L-DOPA, when it produces a significant increase in the "on-time" per day, and reduces response fluctuations. However, there is great interest in the neuroprotective potential of rasagiline, which is currently the objective of a large clinical study (USA Parkinson's Disease Group). Rasagiline may cause neuroprotection by reducing the metabolism of dopamine, but it also possesses its own intrinsic neuroprotective properties.

## Our readers may be unfamiliar with the steps necessary to develop a drug from the lab through testing, approval, sales, and distribution. Can you briefly summarize the process?

Following initial preclinical studies showing potential activity against disease, new drugs are submitted to exhaustive toxicity testing, including long-term administration to several animal species, and studies in pregnant animals. During this period, the preclinical pharmacology studies are also broadened. If all is OK from toxicology, one goes into stage 1 clinical testing in human volunteers to detect any human-selective toxicity, then phase 2 in patients (determination of active dose and maximum-tolerated dose), and full phase 3 study using double-blind design whenever possible. The nail-biting period is while one waits for the results from phase 3, i.e., opening the codes and arranging the data for active and placebo (or comparison drug) groups, since this is the final, objective test of efficacy in humans. If successful, all the data are submitted to the licensing authorities for New Drug Application (NDA).

## What obstacles have you and your colleagues overcome to develop rasagiline as a commercial drug?

Our preclinical studies, for early detection of activity, were carried out in our university labs and in one or two other related institutions. In the case of rasagiline, full clinical development was carried out by Teva, Israel Pharmaceutical Industries Ltd. The fact is that, even if you have proved a drug to be effective clinically, there is still much effort to be invested in order for it to be accepted on the large scale by the medical practitioners around the world. This requires much publicity and marketing, in which medical specialists are invited to join studies with the new drug and to present their data at congresses to ensure that the major clinical centers are positive about the new drug's advantages. The "coming-out party" for rasagiline was at the International Parkinson's Disease Congress in Berlin (2005), and it was a tremendous experience for me to see the rasagiline booth at the commercial exhibition there. Our rasagiline story also appeared at the excellent exhibition on advances in Parkinson's disease at the congress.

## How would you characterize the current paradigms for anti-Parkinson drug discovery?

The major problem in this area, as in most others, is in the choice of the disease model in animals. Parkinson's disease develops over the course of several years, and most patients suffer from the idiopathic disease, for which the cause is unknown. One can reproduce disease symptoms by injecting neurotoxins, but again, the human disease is not caused this way. As a result, the animal models are most useful for studying new ways to reduce symptoms, but are poor predictors of disease-modifying agents.

## What advances do you envision for anti-Parkinson drug discovery in the coming years?

A number of genes whose mutated or overexpressed products cause the genetic forms of the disease have been detected. Although most patients do not suffer from hereditary forms of the disease, study of the causes of inherited parkinsonism is providing clues to the general pathophysiology of the disease. Increasingly, genotyping will be able to predict the at-risk subjects. There will also be advances in early detection, probably by a combination of advanced imaging techniques and genotyping. Early detection is a major problem in Parkinson's disease, since by the time neurological diagnosis is made, more than 50 percent of the substantia nigra neurons have been lost.

## What suggestions for further research would you make?

Almost certainly, Parkinson's disease is caused by a multivalent genetic combination, which may even interact with environmental factors. As a result, it will take a long time before the causative factors are established, and even then, prevention or elimination of this disease may not be possible. Treatments must therefore be further improved, and I believe that cell transplantation therapies will develop in many interesting ways.

## Do you have anything else to add or share?

I feel truly lucky to have been part of this new drug discovery, but there are many other discoveries to come, so keep up the hard work, since there is a good chance to be associated with a major advance, as long as you concentrate on the best quality research.

## Abbreviations

L-DOPA, 3,4-dihydroxy-L-phenylalanine; MAO, monoamine oxidase inhibitor; NDA, new drug application; PD, Parkinson's disease.

## Competing interests

The author(s) declare that they have no competing interests.

**Figure 1:**
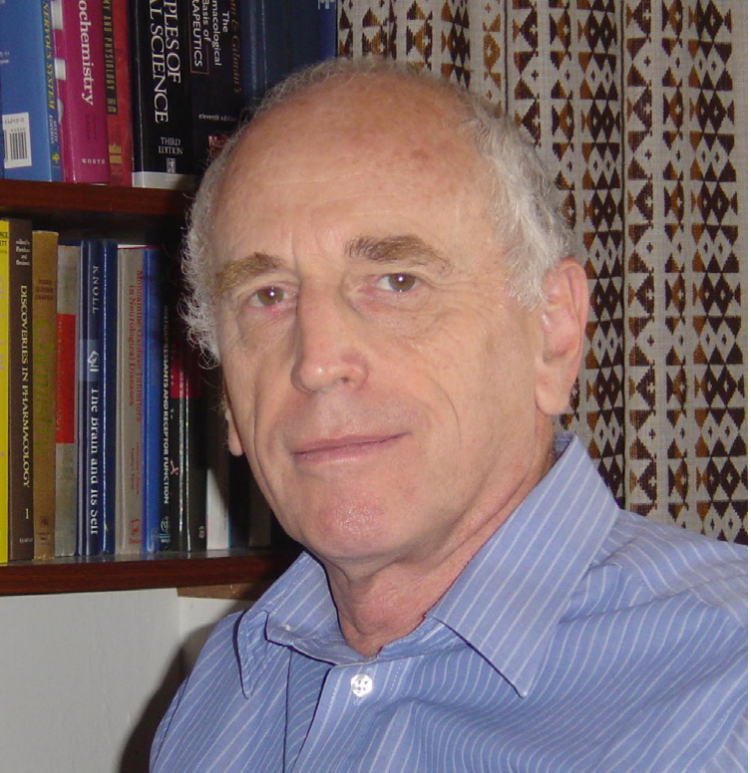
**Photo of Prof. John Fingberg (interviewee)**. Image provided by Finberg.

## References

[B1] Finberg JPM, Pacak K, Kopin IJ, Goldstein DS (2003). Chronic inhibition of monoamine oxidase type A increases noradrenaline release in rat frontal cortex. Naunyn-Schmiedeberg's Arch Pharmacol.

[B2] Finberg JPM, Tenne M (1982). Relationship between tyramine potentiation and selective inhibition of MAO types A and B in the rat vas deferens. Brit J Pharmacol.

[B3] Lamensdorf I, Youdim MBH, Finberg JPM (1996). The effect of long term treatment with monoamine oxidase A and B inhibitors on dopamine release from rat striatum in vivo. J Neurochem.

[B4] Finberg JPM, Takeshima T, Johnston JM, Commissiong JW (1998). Increased survival of dopaminergic neurons by rasagiline, a monoamine oxidase B inhibitor. Neuroreport.

[B5] Bonneh-Barkay D, Ziv N, Finberg JPM (2004). Characterisation of neuroprotective effect of rasagiline in cerebellar granule cells. Neuropharmacology.

[B6] Finberg JPM, Sader-Mazbar O (2007). Modification of L-DOPA pharmacological activity by MAO inhibitors. J Neural Transm.

